# Effect of *Lampaya medicinalis* Phil. (Verbenaceae) and Palmitic Acid on Insulin Signaling and Inflammatory Marker Expression in Human Adipocytes

**DOI:** 10.3390/ph17050566

**Published:** 2024-04-29

**Authors:** Gabriela Yuri, Mariana Cifuentes, Pedro Cisternas, Adrián Paredes, Paulina Ormazabal

**Affiliations:** 1 Institute of Health Sciences, Universidad de O’Higgins, Av. Libertador Bernardo O’Higgins 611, Rancagua 2820000, Chile; gabriela.yuri@inta.uchile.cl (G.Y.); pedro.cisternas@uoh.cl (P.C.); 2 Laboratory of Obesity and Metabolism in Geriatrics and Adults (OMEGA), Institute of Nutrition and Food Technology (INTA), Universidad de Chile, Av. El Líbano 5524, Macul, Santiago 7830490, Chile; mcifuentes@inta.uchile.cl; 3 Advanced Center for Chronic Diseases (ACCDiS), Santiago 8380453, Chile; 4 Laboratorio de Química Biológica, Instituto Antofagasta (IA) and Departamento de Química, Facultad de Ciencias Básicas, Universidad de Antofagasta, Av. Angamos 601, Antofagasta 1240000, Chile; adrian.paredes@uantof.cl; 5 Escuela de Obstetricia, Facultad de Ciencias para el Cuidado de la Salud, Universidad San Sebastián, Santiago 8330106, Chile

**Keywords:** adipocyte, palmitic acid, insulin signaling, glucose uptake, inflammation, *Lampaya medicinalis*

## Abstract

Background: Aging and obesity are associated with insulin resistance (IR) and low-grade inflammation. Molecularly, IR is characterized by a reduction in glucose uptake and insulin signaling (IRS-1/Akt/AS160 pathway), while inflammation may result from upregulated NF-κB pathway after low Tyr-IκBα phosphorylation. Upregulated phosphatase activity of PTP1B is associated with impaired insulin signaling and increased inflammation. Plasma levels of palmitic acid (PA) are elevated in obesity, triggering inflammation and disruption of insulin signaling. Traditional medicine in Northern Chile uses oral infusions of *Lampaya medicinalis* Phil. (Verbenaceae) to treat inflammatory conditions. Significant amounts of flavonoids are found in the hydroethanolic extract of Lampaya (HEL), which may account for its biological activity. The aim of this work was to study the effect of HEL and PA on insulin signaling and glucose uptake as well as inflammatory marker expression in human adipocytes. Methods: We studied HEL effects on PA-induced impairment on insulin signaling, glucose uptake and inflammatory marker content in human SW872 adipocytes. HEL cytotoxicity was assessed in adipocytes at different concentrations (0.01 to 10 g/mL). Adipocytes were incubated or not with PA (0.4 mM, 24 h) with or without HEL (2 h pre-incubation), and then stimulated with insulin (10 min, 100 mM) or a vehicle. Phospho-IRS-1, phospho-Akt, phospho-AS160, phospho-NF-κB and phospho-IκBα, as well as protein levels of PTP1B, were assessed using Western blotting, and glucose uptake was evaluated using the 2-NBDG analogue. Results: At the assessed HEL concentrations, no cytotoxic effects were observed. PA decreased insulin-stimulated phospho-Akt and glucose uptake, while co-treatment with HEL increased such markers. PA decreased phospho-IRS-1 and phospho-Tyr-IκBα. On the other hand, incubation with HEL+PA decreased phospho-AS160 and phospho-NF-κB compared with cells treated with PA alone. Conclusion: Our results suggest a beneficial effect of HEL by improving PA-induced impairment on molecular markers of insulin signaling, glucose uptake and inflammation in adipocytes. Further studies are necessary to elucidate whether lampaya may constitute a preventive strategy for people whose circulating PA levels contribute to IR and inflammation during aging and obesity.

## 1. Introduction

Globally, the population 65 years of age and above, who are more likely to have chronic conditions, is expected to rise from 761 million in 2021 to 1.6 billion in 2050 [1,2]. Since the current pandemic of obesity worsens age-related deterioration of tissue function [3], many efforts worldwide are being made to achieve healthy aging [4]. Both obesity and aging are associated with the development of metabolic alterations [5,6]. Obesity is defined as an excess of body fat, and leads to a wide range of pathologies such as dyslipidemia, cancer, cardiovascular disease, insulin resistance (IR) and type 2 diabetes mellitus (T2DM), which are age-related disorders [5,6,7,8]. Indeed, aging individuals with obesity may be more susceptible to long-term chronic health complications; therefore, there is a need to seek pharmacological and lifestyle interventions to prevent aging and obesity-related pathologies [9].

White adipose tissue (WAT) is the main reservoir of energy. In advanced states of obesity, excess triglycerides are stored in different organs as ectopic fat, producing oxidative stress and inflammation [6,10]. At a cellular level, obesity is characterized by altered cell signaling in the adipocyte, i.e., a malfunctioning of the insulin receptor (INSR)/IRS-1/Akt/AS160 pathway activation, which may result, among others, in reduced insulin-induced glucose uptake [11,12]. There is increasing evidence linking obesity and both local and systemic inflammation. Chronic inflammation and IR have been linked in both correlational and causal studies in human populations and animal models [13]. Among insulin signaling regulators, protein tyrosine phosphatase 1B (PTP1B) removes Tyr phosphates from the INSRβ and IRS-1, attenuating insulin signaling transduction [1,14,15]. Interestingly, the tyrosine phosphatase PTP1B can dephosphorylate phospho-Tyr42 IκBα, which favors its activation by phosphorylation at Ser. In turn, activated IκBα is associated with inflammation upregulation by increasing the activity of a key proinflammatory factor, NF-κB [16].

Circulating saturated fatty acids, including palmitic acid (PA), are elevated in individuals with obesity. This situation is exacerbated by the high dietary concentration of this fatty acid in Western diets [17,18,19]. Elevated serum concentrations of SFAs have been associated with the development of metabolic conditions induced by obesity, including IR and chronic inflammation [20,21]. Evidence from in vitro studies shows that PA exposure reduces the phosphorylation of IRS-1, Akt and AS-160 in addition to inhibiting insulin-induced glucose uptake in 3T3-L1 adipocytes [22]. On the other hand, cultured cells exposed to elevated PA show increased levels of PTP1B as well as activation NF-κB [23,24]. Thus, the implementation of new therapeutic approaches to prevent or counteract the obesity-associated inflammation and disrupted insulin signaling is encouraged, especially given that the world’s population is aging rapidly [25,26]. The use of medicinal plants against obesity-related diseases is gaining increased attention [27]. *Lampaya medicinalis* Phil. (Verbenaceae), also known as “lampaya”, is a small bush that grows in the “Puna atacameña” in northern Chile [28,29]. According to oral tradition, boiled water infusions of leaves from the plant are used to treat stomach pain, colds, cough, urinary bladder discomforts, arthritis, rheumatism and joint pain [30]. The in vitro anti-inflammatory and antioxidant properties of Lampaya hydroethanolic extract (HEL) [28,29,31,32] might be explained by its high concentrations of phenols and flavonoids, as well as minor amounts of naphthalenic and iridoid glycosides, phenolic acid and derivatives of p-hydroxyacetophenones [32].

Interestingly, in vitro exposure to HEL improves the PA-induced proinflammatory response in human macrophages, the PA-induced impairment of the IRS-1/Akt/AS160 pathway and the ability of murine adipocytes to incorporate glucose intracellularly [32,33]. However, it is unknown whether HEL improves insulin signaling and glucose uptake, as well as inflammatory marker expression in human adipocytes. Thus, given that adipose tissue is a target for therapeutic intervention in obesity and age-related diseases, in this work, we examined the effect of HEL in PA-treated human SW872 adipocytes by assessing insulin signaling, glucose uptake and inflammatory marker content.

## 2. Results

### 2.1. Adipogenic Differentiation in SW872 Cells

**Figure 1:**
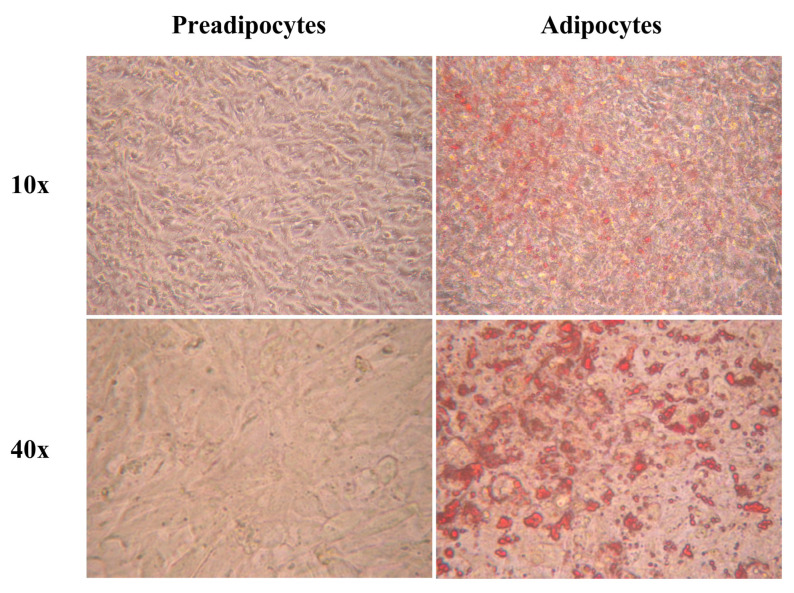
SW872 preadipocytes and adipocytes stained with Oil Red O. SW872 cells were differentiated for ten days as indicated in the Methods section. The red staining shows triglyceride accumulation. Total magnification: 100× and 400×.

Oil Red O staining was used to determine preadipocyte differentiation into adipocytes. Red staining of intracellular lipid droplets in SW872 adipocytes revealed triglyceride accumulation (Figure 1). SW872 preadipocytes did not exhibit intracellular red staining.

After 10 days of adipogenic differentiation, the phenotype of mature adipocytes was confirmed.

### 2.2. The Hydroethanolic Extract of Lampaya (HEL) Is Not Cytotoxic on SW872 Adipocytes

**Figure 2:**
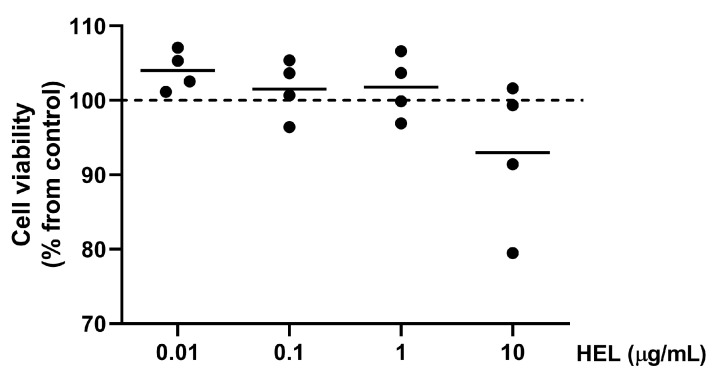
Cell viability in in vitro differentiated SW872 adipocytes exposed to different concentrations of HEL. MTS assay was conducted on cells treated with HEL at 0.01, 0.1, 1.0 and 10.0 µg/mL for 26 h. Results are presented as percentages relative to untreated cells, shown as the dotted line at 100% (control). Each independent experiment (conducted in triplicate) is represented as a dot, while the line represents the mean.

To evaluate any possible cytotoxic effects of HEL on SW872 cells, adipocytes were exposed to various concentrations of HEL (0.01, 0.1, 1 and 10 µg/mL) for 26 h before the MTS assay was carried out. Compared to the control, HEL exhibited no significant impact on cell viability, despite high data variability at 10 µg/mL (Figure 2). As a result, the lowest concentration of HEL (0.01 μg/mL) was selected for the experiments.

### 2.3. HEL Restores Impaired IRS-1 and Akt Phosphorylation Induced by PA in SW872 Adipocytes

**Figure 3:**
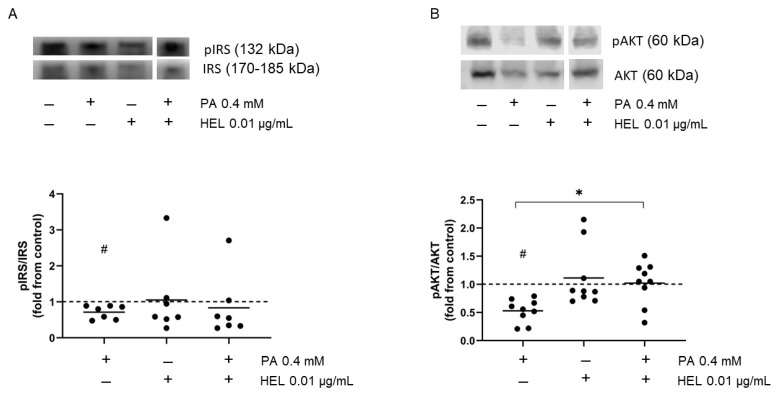

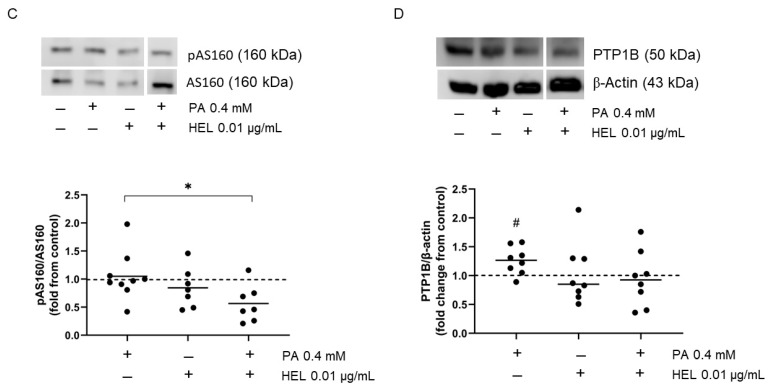
HEL restores the PA-induced impairment in insulin-stimulated phosphorylation of IRS and Akt in SW872 cells. Cells were treated with PA (0.4 mM, 24 h), with or without HEL (0.01 µg/mL, 26 h). Adipocytes were exposed to insulin [(**A**–**C**)] (100 mM, 10 min) or not (**D**). Immunoblot images show phosphorylation of (**A**) IRS-1, (**B**) Akt, (**C**) AS160 and protein levels of (**D**) PTP1B in SW872 adipocytes. The results are shown as fold change from control and the dotted line indicates the control (value of 1). Each value on the graph is represented by a dot, and the mean from n = 7–9 independent experiments is shown by a line. # *p* < 0.05 for the difference from vehicle-treated cells, * *p* < 0.05 for the difference between PA and HEL+PA treated cells. Student’s *t* test or generalized linear model, according to data distribution.

In order to assess the impact of HEL on insulin signaling activation in SW872 adipocytes, phospho-IRS-1, phospho-Akt and phospho-AS160 were evaluated. As Figure 3A–C shows, the exposure to PA reduced insulin-stimulated phosphorylation of IRS-1 (28%, *p* < 0.05, Figure 3A) and Akt (47%, *p* < 0.05, Figure 3B), as compared to untreated cells, respectively. PA did not change AS160 phosphorylation levels compared to control cells (Figure 3C). On the other hand, PA increased PTP1B protein levels, as compared to control cells (26%, *p* < 0.05, Figure 3D). To assess the role of HEL on PA-impaired insulin signaling, we examined the levels of phosphorylated IRS-1, Akt and AS160, as well as the protein expression of PTP1B in adipocytes co-treated with HEL. To this end, 0.01 µg/mL HEL was administered 2 h prior to, and maintained throughout, the exposure with PA (24 h). The presence of HEL partially restored PA-induced reduction in Akt phosphorylation (92%, *p* < 0.05, Figure 3B), suggesting the ability of the extract to reverse the change induced by PA. Phospho-AS160 remained unchanged after PA- treatment in adipocytes; however, co-treatment with HEL+PA diminished such phosphorylation (48%, *p* < 0.05, Figure 3C). No differences were observed for phospho-IRS-1 and PTP1B protein levels between adipocytes treated with PA and HEL+PA cells (Figure 3A,D). When compared to vehicle-treated cells, HEL alone had no effect on insulin-stimulated phosphorylation of IRS-1, Akt, AS160 or PTP1B (Figure 3A–D).

### 2.4. HEL Counteracts Palmitic Acid-Impaired Glucose Uptake in SW872 Adipocytes

Given that glucose uptake serves as a critical endpoint for metabolic insulin signaling in adipocytes, we evaluated whether HEL modulates this phenomenon in PA-treated cells.

**Figure 4:**
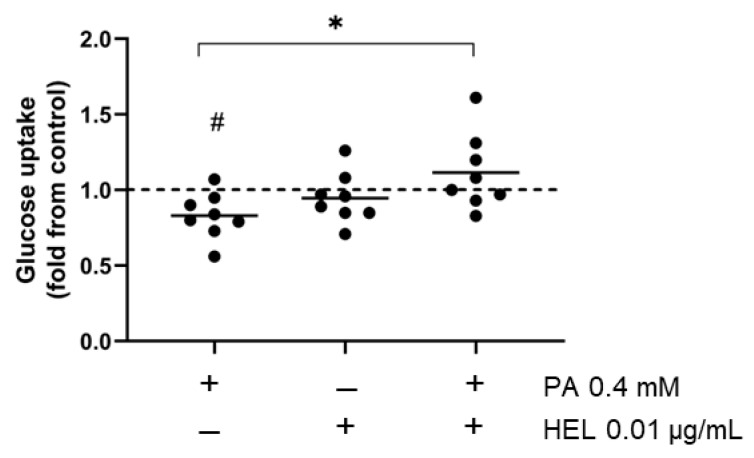
HEL restores the PA-induced decrease in glucose uptake in SW872 cells. Cells were treated with PA (0.4 mM, 24 h), and with or without HEL (0.01 µg/mL, 26 h). The fluorescent glucose analog 2-NBDG was used to assess insulin-stimulated glucose uptake, as stated in the Methods section. The results are shown as fold change and the dotted line indicates the control (value of 1). The graph displays individual data points as dots, with the mean indicated by a line (n = 8). # *p* < 0.05 for the difference from vehicle-treated cells, * *p* < 0.05 for the difference between PA and HEL+PA treated cells. Student’s *t* test or generalized linear model, according to data distribution.

As expected, glucose uptake decreased after PA exposure (17%, *p* < 0.05) compared to untreated cells. Interestingly, HEL abolished PA-induced reduction in glucose uptake in adipocytes (35%, *p* < 0.05, Figure 4). When compared to vehicle-treated cells, HEL had no effect on insulin-stimulated glucose uptake (Figure 4).

### 2.5. Effect of HEL on IκBα/NF-κB Signaling in PA-Treated SW872 Adipocytes

**Figure 5:**
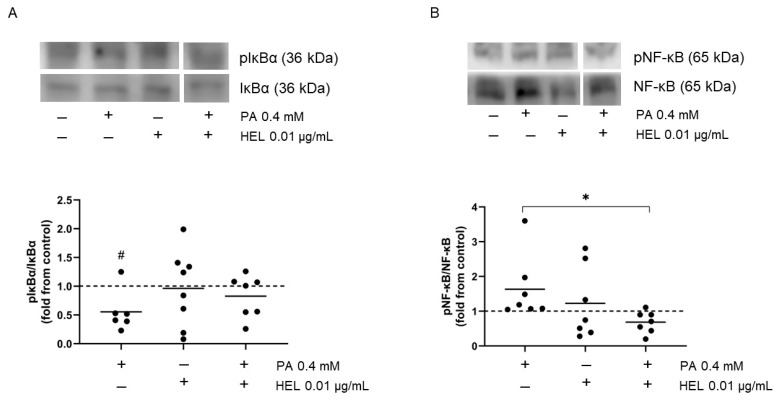
Effect of HEL on the phosphorylation of Tyr42-IκBα and NF-κB in SW872 adipocytes. Cells were treated with PA (0.4 mM, 24 h), with or without HEL (0.01 µg/mL, 26 h). Immunoblot images show phosphorylation of (**A**) IκBα and (**B**) NF-κB in SW872 adipocytes. The results are shown as fold change and the dotted line indicates the control (value of 1). Each value on the graph is represented by a dot, and the mean from n = 6–7 independent experiments is shown by a line. # *p* < 0.05 for the difference from vehicle-treated cells, * *p* < 0.05 for the difference between PA and HEL+PA treated cells. Student’s *t* test or generalized linear model, according to data distribution.

Considering that PA increased PTP1B content (Figure 3D) and that PTP1B dephosphorylates phospho-Tyr42-IκBα, leading to inflammation by upregulating the NF-κB pathway, we addressed whether PA and HEL could modulate Tyr42-IκBα and NF-κB phosphorylation in SW872 adipocytes. Interestingly, PA reduced the phosphorylation of IκBα as compared to untreated cells (45%, *p* < 0.05, Figure 5A), whereas there were no differences for phospho-Tyr42-IκBα between HEL+PA and PA- treated adipocytes.

In comparison to untreated cells, PA had no effect on the phosphorylation of NF-κB (Figure 5B). Nevertheless, reduced phospho-NF-κB was present in cells incubated with HEL+PA compared to SFA-treated adipocytes (42%, *p* < 0.05, Figure 5B). Compared to vehicle-treated cells, HEL had no effect on the phosphorylation of IκBα and NF-κB (Figure 5).

## 3. Discussion

Obesity occurs when an excessive or abnormal fat accumulation can be detrimental to health, and is considered an important risk factor for IR, T2DM, cardiovascular disease and some types of cancer [34]. Functional changes in WAT occurring in obesity, are also a feature of aging [35]. Interestingly, chronic inflammation in obesity plays a critical role in the development of IR [10]. Due to the increase in the prevalence of obesity and its comorbidities, alternative therapies have been sought for the treatment or prevention of these pathologies [36,37,38]. Ancestrally, the population has used medicinal plants to treat or cure diseases. Interestingly, oral reports in Chile indicate that lampaya infusion is used for the treatment of some inflammatory diseases [30].

Previous work from our group has assessed the role of HEL on FA-induced proinflammatory and fibrosis markers in human macrophages and hepatocytes [31,32]. Evidence regarding the role of *Lampaya medicinalis* on PA-disrupted insulin signaling and glucose uptake as well as inflammatory molecules in human adipocytes was lacking. SW872 is a human model of preadipocytes with the ability to differentiate into mature adipocytes. These cells have been employed in vitro to study adipose tissue dysfunction associated metabolic conditions such as obesity [39]. The effect of *Lampaya medicinalis* in PA-treated SW872 adipocytes is demonstrated for the first time in this in vitro study. According to our findings, HEL counteracts PA-impaired Akt activation and glucose uptake, and it regulates NF-κB phosphorylation in the presence of PA. HEL did not affect the viability of SW872 cells, which agrees with previous reports from our group showing the lack of cytotoxic effects of HEL, at the ranges assessed here, on different cell types [31,33]. Evidence from murine adipocytes has shown the ability of HEL to restore of PA- impaired phospho-Akt, phospho-IRS-1, phospho-AS160, phospho-NF-κB and glucose uptake [33]. Here, a beneficial role was also evidenced in a human adipose cell line, agreeing with the documented protective effect of HEL against PA-induced molecular impairment found in human macrophages and hepatocytes [31,32].

Bioactive compounds derived from plants, such as polyphenols, are gaining popularity due to their ability to improve the insulin cascade [28]. *Lampaya medicinalis* has been used as an infusion in traditional herbal medicine to treat a variety of health conditions. The efficiency of herbal infusions is most likely due to the synergy of multiple components rather than the activity of a single molecule [40]; therefore, it is crucial to note that we chose to examine HEL as a whole rather than just its individual and pure components.

Since the solvents and methods used to extract plant metabolites determine the specific phytochemical content, it is improbable that constituents obtained in HEL resemble those found in lampaya aqueous extractions. We chose to focus on HEL and not any other fractions from the *Lampaya medicinalis* extracts because ethanolic extractions primarily contain and concentrate molecules with anti-inflammatory and antioxidant effects, which are associated beneficial activities against chronic conditions associated with aging, while water extracts primarily contain compounds stimulating the immune system [41,42,43].

HEL is a phenol-rich mixture containing twenty-three molecules, comprising one naphthalenic and iridoid glycoside, five phenolic acid derivatives, two acetophe-none derivatives and fourteen flavonoid derivatives [32]. HEL has shown anti-inflammatory and antioxidant activities in vitro and in vivo [28,29,32,33]. Phenols and flavonoids are well known to improve insulin responsiveness in vivo and in vitro [44,45,46]. In this study, HEL counteracts PA-induced downregulated Akt activation and glucose uptake in SW872 adipocytes. Thus, such findings may be explained by the phytochemical composition of HEL.

PA, a proinflammatory SFA that is elevated in individuals with obesity, is associated with IR [47]. In the present study, PA decreased insulin-induced phosphorylation of IRS-1 and Akt as well as glucose uptake in SW872 adipocytes. Interestingly, impaired phospho-Akt and glucose uptake were abrogated by HEL. Licarin B, a neolignan isolated from *Myristica fragrans,* increased GLUT4 expression and translocation through the IRS-1/PI3K/AKT pathway, leading to enhanced insulin sensitivity [48]. Interestingly, extracts from *Tetrapleura tetraptera, Aframomum melegueta* and *Zanthoxylum leprieurii* have shown to increase glucose uptake in SW872 human adipocytes [49]. Even though HEL alone did not upregulate glucose uptake in SW872 adipocytes, it showed a beneficial effect preventing its impairment by PA in SW872 cells.

PTP1B is a phosphatase that dephosphorylates the insulin receptor and post-insulin receptor substrates such as IRS-1, impairing insulin signaling [50,51]. To our knowledge, no data regarding the effect of PA on PTP1B in human adipocytes is available, however, evidence indicates that treatment with PA up-regulated PTP1B in rat skeletal muscle cells (L6 myotubes) [52], while an extract of *Artemisia dracunculus* L. is able to abolish such increment [50]. Our data showed no effect of HEL preventing PA-induced increase in PTP1B levels in SW872 adipocytes. Due to the ability of PTP1B to remove Tyr phosphates from IRS-1 [14,15], the increased expression of PTP1B after PA exposure may be related with the low Tyr-phosphorylation levels of IRS-1 found in SW872 adipocytes treated with the SFA, nevertheless, additional research is required to determine the precise causal relationships between PTP1B activity and IRS-1/Akt route activation.

On the other hand, it is well known that PA induces inflammation by activating the NF-κB pathway [53]. As far as we know, this is the first report that highlights the effect of PA on Tyr-IκBα phosphorylation in human adipocytes. Here we evidence a lower phosphorylation of IκBα after treatment with PA, which might be related with the high content of PTP1B found in PA- treated cells. However, more studies are required to determine the role of PTP1B on phospho-Tyr-IκBα in SW872 adipocytes. Our findings evidence a downregulation of NF-κB phosphorylation in PA+HEL-treated adipocytes, compared to cells treated with the SFA alone. Similarly, flavonoid-rich plant extracts have been shown to counteract NF-κB phosphorylation in 3T3-L1 adipocytes [53,54].

In conclusion, HEL counteracts PA-impaired insulin-stimulated activation of Akt and glucose uptake, and it downregulates NF-κB activation in human SW872 adipocytes. We suggest that the flavonoid content of HEL has an impact on PA-treated adipocytes’ Akt and NF-κB phosphorylation and glucose uptake. However, more studies are required to determine the contribution of HEL on SFA- modulation of insulin responsiveness and inflammatory response, phenomena that become impaired in obesity and throughout the aging process. Future investigations, particularly in vivo studies, should be conducted to determine the effectiveness and biological mechanism of HEL against IR and inflammation. Furthermore, the assessment of the bioavailability of HEL components as well as its safety and effectiveness in human populations should be considered in order to propose HEL as a therapeutic tool in obesity and aging-related conditions.

## 4. Materials and Methods

### 4.1. Herb Material and HEL Preparation

*Lampaya medicinalis* Phil. leaves and aerial parts were taken in Socaire, Northern Chile (23°36′40 s S; 67°50′33 s W, 3230 m above sea level). The vegetal material was recognized by scientific personnel, and voucher samples are housed in the Herbarium of Universidad de Concepción, Chile.

*Lampaya medicinalis* Phil. air-dried leaves (1.2 kg) were mechanically ground to obtain a fine powder. The powder was then placed in a cotton bag and left to be extracted with a combination of EtOH:H_2_O (1:1, 10 L) thoroughly for a week at room temperature. The EtOH:H_2_O extract was filtered and evaporated under reduced pressure to eliminate the ethanol. A Labconco 4.5 FreeZone lyophilizer was used to freeze dry the aqueous extract, resulting in a viscous dark green mass (HEL). The lyophilized solution yielded 12.5% (*w*/*w*). The dried extract was sealed in a bottle and stored at 4 °C until it was needed. Just prior to use, the extract was diluted to create a 10 mg/mL stock solution using dimethyl sulfoxide (DMSO, Sigma-Aldrich, St. Louis, MO, USA). Additional dilutions of the stock in culture media were prepared.

### 4.2. Culture and Differentiation of SW872 Preadipocytes

The cell line SW872 (ATCC HTB-92, Manassas, VA, USA) derived from a human fibrosarcoma was used. Preadipocytes were grown in an incubator with a controlled atmosphere (5% CO_2_) at 37 °C in Dulbecco′s Modified Eagle′s Medium/Nutrient Mixture F-12 Ham (DMEMF-12, Sigma-Aldrich, St. Louis, MO, USA) supplemented with 10% fetal bovine serum (FBS, Biological Industries, Beit-Haemek, Israel) and antibiotics (Penicillin-Streptomycin, Biological Industries, Beit-Haemek, Israel). Preadipocytes were cultured in DMEMF-12 with 1% FBS for 10 days after confluence and supplemented with 1 M dexamethasone (Sigma-Aldrich, St. Louis, MO, USA), 1 M rosiglitazone (Calbiochem^®^, Darmstadt, Germany), 10 g/mL insulin (Insuman^®^, Sanofi-Aventis, Paris, France) and 0.5 mM 1-methyl-3-isobutyl-xanthine (Calbiochem^®^, Darmstadt, Germany).

Cells were fixed with 4% formaldehyde and stained with the lipophilic dye Oil Red O (Sigma–Aldrich, St. Louis, MO, USA) to confirm fully mature adipocyte phenotype.

### 4.3. Cell Viability and Treatments

The viability of adipocytes exposed to different concentrations of HEL was measured using a cell proliferation assay (CellTiter 96^®^ Aqueous One Solution Cell Proliferation Assay, Promega, Madison, WI, USA) following the manufacturer’s instructions to determine the concentration of HEL to be used in this study. Cells were incubated with 0.01, 0.1, 1 and 10 μg/mL of HEL for 26 h after differentiation (day 10). Following treatments, 20 µL of 3-(4,5-dimethylthiazol-2-yl)-5-(3-carboxymethoxyphenyl)-2-(4-sulfophenyl) were added. After 3 h, an ELx808 microplate reader (BioTek Instruments, Inc. Winooski, VT, USA) was used to measure absorbance (A) at 490 nm. Background absorbance at 630 nm was measured and subtracted to A_490 nm_. Results were presented as percentage of control cells.

We used 10% FFA-Bovine Serum Albumin (Sigma-Aldrich, St. Louis, MO, USA) to prepare PA (Sigma-Aldrich, St. Louis, MO, USA)). After differentiation was completed, cells were exposed to PA and/or HEL, and then acutely stimulated with insulin. Then, differentiated SW872 adipocytes were incubated with PA or vehicle for 24 h with or without HEL (2 h preincubation) and then stimulated or not with insulin. Therefore, experimental conditions were as follows: untreated cells (control), 0.4 mM PA, 0.01 μg/mL of HEL, 0.01 μg/mL of HEL (2 h before) + 0.4 mM PA, in basal or insulin-stimulated (100 mM, 10 min) conditions. Concentration and exposure time of insulin were those previously described to produce a two-fold increase in the phosphorylation of Akt compared to non-stimulated adipocytes [55]. Insulin signaling and glucose uptake was assessed in insulin-stimulated adipocytes while inflammatory markers were evaluated in basal condition.

### 4.4. Western Blotting

Total cell lysates were obtained by sonicating SW872 cells at 4 °C in lysis buffer (50 mM Tris base, 150 mM NaCl, 10 mM Sodium Pyrophosphate, 100 mM NaF, 2 mM Ortovanadate, 1% NP40, pH 8.0, plus PhosSTOP (Roche, Mannheim, Germany)). Cell lysate protein concentration was measured using a bicinchoninic acid technique (Pierce, Rockford, IL, USA). A total of 40 micrograms of protein was heat denatured in SDS-PAGE loading buffer (240 mM Tris-HCl, 40% glycerol, 8% SDS, 20% 2-mercaptoethanol, pH 6.8). Electrophoresis of proteins was performed on 8–10% polyacrylamide gels and then gels were electrotransferred to 0.22 m nitrocellulose membranes (AmershamTM Protran^®^, Munich, Germany) in a cold solution containing 194 mM glycine, 24 mM Tris and 20% methanol. Membranes were blocked with a 5% BSA (Sigma-Aldrich, St. Louis, MO, USA) solution in Tris-buffered saline (TBS) with 0.05% Tween 20 (Sigma-Aldrich, St. Louis, MO, USA)) and subsequently the immunoreaction was achieved by incubating the membranes with antibodies against anti-IRS-1 (E-12) (1:300; Santa Cruz Biotechnology, Dallas, TX, USA), phospho-IRS1 (Tyr612) (1:500; Invitrogen, Waltham, MA, USA), anti-IκBα (1:300; Cell Signaling, Danvers, MA, USA), anti-IκBα (1:300; Cell Signaling, Danvers, MA, USA), anti-phospho IκBα (Tyr42) (1:200; Invitrogen, Waltham, MA, USA), anti-Akt (1:1000; Cell Signaling, Danvers, MA, USA), anti-phospho-Akt (Ser473) (1:1000, Cell Signaling, Danvers, MA, USA), anti-NF-κB p65 (D14E12) (1:200; Cell Signaling, Danvers, MA, USA), anti-phospho-NF-κB p65 (S536) (1:200; Cell Signaling, Danvers, MA, USA), anti-PTP1B (D-4) (1:1000; Santa Cruz Biotechnology, Dallas, TX, USA), AS160 (C69A7) (1:500; Cell Signaling, Danvers, MA, USA), anti-phospho AS160 (Thr642) (1:500, Cell Signaling, Danvers, MA, USA). As an internal loading control, anti-actin (1:3000; Santa Cruz Biotechnology, Dallas, TX, USA) was used. Once membranes were incubated with peroxidase-conjugated secondary antibodies, an incubation with enzyme substrates (Westar Supernova (Cyanagen, BO, Italy)) was followed to detect the immune complexes by using the gel documentation system LI-COR C-DiGit Blot Scanner (Lincoln, NE, USA). ImageJ software version 1.53 h (NIH, Bethesda, MD, USA) was used to digitize the images and quantify the band densities.

### 4.5. Glucose Uptake

The glucose uptake assay was conducted following the methodology outlined by Yamamoto et al. [56] with some modifications. Equivalent quantities of SW872 preadipocytes were plated in 6-well dishes, and upon reaching confluence, the cells underwent differentiation as described above. After 10 days of differentiation, adipocytes were treated or not with 0.4 mM PA for 24 h in the presence or absence of HEL as previously outlined. Once the treatments were concluded, adipocytes were washed and subjected to a 2 h serum starvation period in DMEM (Sigma–Aldrich, St. Louis, MO, USA). Following this, the medium was removed, and cells were washed two times with Kreb’s Ringer Buffer without glucose (KRB w/o glu) supplemented with 145 mM NaCl, 10 mM HEPES (pH 7.4), 2.6 mM CaCl_2_, 5 mM KCl, 1 mM MgCl_2_. Subsequently, the adipocytes were stimulated with 100 mM insulin in KRB w/o glu for 30 min at 37 °C in a 5% CO_2_ atmosphere. After cell wash, a fluorescent glucose analogue, 2-(N-(7-nitrobenz-2-oxa-1,3-diazol-4-yl) amino)-2-deoxyglucose (2-NBDG, 300 μM) (Cayman Chemical, Ann Arbor, MI, USA) was added, and the cells were subsequently incubated for an additional 15 min. The uptake of the fluorescent analogue was stopped by aspirating the incubation medium and cells were washed twice with KRB w/o glu. Lysis was carried out by adding to the wells a buffer containing 150 mM NaCl, 50 mM Tris base, 1% NP40, pH 8.0, and lysates were collected and placed in a black, clear-bottomed 96-well culture plate. Fluorescence was measured at 465/540 nm (wavelength excitation/emission) using a fluorimeter (Synergy 2 fluorimeter, BioTek Instruments Inc. Winooski, VT, USA).

### 4.6. Statistical Analysis

The distributional normality of the data was determined using the Shapiro–Wilk test. To assess differences in parameters between two conditions, we employed Student’s *t* test. Non-normal distributions were adjusted to a gamma distribution and, consequently, comparison between conditions was evaluated by probability distribution using a generalized linear model. STATA version 18.8 software was used for the analysis. The data is shown as a dot plot with mean. A *p*-value < 0.05 was considered significant.

## Data Availability

Data is contained within the article.
